# Swedish managers’ experience of yearly staff development dialogues, aiming for employee development, performance, and well-being

**DOI:** 10.1186/s40359-022-00890-w

**Published:** 2022-07-27

**Authors:** Åsa Bringsén, Petra Nilsson Lindström

**Affiliations:** Faculty of Health Sciences, Kristianstad University, 291 88 Kristianstad, Sweden

**Keywords:** Health management, Focus groups, Managers, Performance, Staff development dialogue, Wellbeing, Health promotion

## Abstract

**Background:**

Societal development and a competitive corporate climate have resulted in increased emphasis on performance management (PM) but also sustainability and health challenges in working life. Opportunities for employee well-being, development and performance are thus highly relevant for organisations and society. PM includes a manager-employee dialogue process and is identified as a complex challenge when combined with employee development and well-being. Managers have a key role in PM, and research in collaboration with practitioners in specific contexts is needed. An exploratory study, focusing on managers’ experience of dialogues between managers and employees in practice, was therefore conducted in collaboration with an inter-municipal corporation company in southern Sweden.

**Methods:**

A qualitative study with an inductive design, supported by semi-structured focus group interviews, was chosen based on the exploratory character of the study. Thirty-five managers were approached in the company and 15, of varying age and managerial experience, agreed to participate. Staff Development Dialogues (SDD) were used in the company PM model to facilitate employee development, performance, and well-being. Data was analysed using conventional qualitative content analysis to obtain new insights without using pre-set classifications.

**Results:**

The analysis resulted in three categories of SDD experiences: SDD in a business context; Managers in relation to SDD; and Employees in relation to SDD. The findings revealed varying SDD experiences as well as approaches, and analytical themes were considered in relation to the organisational context and the specific SDD content and process. The study showed the importance of SDD adaptation and a well-being perspective in the dialogues was related to relational aspects and the manager-employee approach to SDDs.

**Discussion:**

The findings confirm the complexity and challenges of PM including development and well-being. Adaptation to specific departments as well as employees is important, and using the same PM strategy throughout an organisation can be questioned. The identified link between a relational approach and a well-being perspective indicates a missed opportunity for systematic workplace health promotion. Strengthening the well-being perspective can, thus, improve the quality of an SDD model, which in turn can facilitate the creation of sustainable workplaces and better fulfilment of employers’ health-related obligations for systematic work environment management.

**Supplementary Information:**

The online version contains supplementary material available at 10.1186/s40359-022-00890-w.

## Background

Technological development, innovation and globalisation have resulted in changes of practice for the adult population in working life, with adherent opportunities and challenges on organisational as well as individual levels. Recognition of human capital as a resource has been identified as the most important strategic factor for successful coping with changes and global competition [1]. The changes and challenges in working life together with a competitive corporate climate have resulted in increased emphasis on performance management (PM), including employee performance assessment (PA) and improvement [2]. PM and PA are related but not identical concepts [2, 3]. PA is described as a formal dialogue process where employees’ performance at work is valued and rated with support from a predetermined set of indicators [2]. The outcome of PA is then often used for setting a performance related individual salary for each employee and can also be used in decisions regarding for instance employee promotions or sanctions [2, 4, 5]. The purpose of PA is to stimulate employees to continuously improve their performance at work [4–6], but yearly meetings characterised by PA have previously been criticised for being inefficient [7–9]. Research has shown that ineffective PA can result in numerous negative consequences, related to the employees and managers as well as the organisation [10]. A well-functioning PA can, on the other hand, result in improved performance and job satisfaction [4, 11, 12]. An increased focus on development and motivation for performance, over the years, can be related to the broadening of the performance perspective through the concept of PM [2].

Thus, PM is described as a broader concept including more and varying procedures, policies and activities [2, 3]. PM programmes or models usually start with PA but focus more on employee performance and development, in relation to organisational performance and strategic goals (2). Both individual and organisational aspects are thus part of an action-oriented cyclic process of PM that aims to improve organisational effectiveness, decision-making and strategy planning (2,3). PM is a complex phenomenon which has been studied without a joint theoretical or practical frame of reference [3]. However, a recently conducted review of PM research resulted in a systems-based taxonomic model showing that PM consists of six components in total, represented by: Inputs, Outputs and the four interdependent process factors, namely Tasks, Individuals, Formal and Informal processes [3]. The core of the model is constituted of the seven interrelated performance tasks: deciding on performance expectations, observing employee performance, integrating performance information, generating a formal summative performance evaluation, delivering performance feedback, the formal performance review meeting, and performance coaching. Formal and informal processes surround and relate to the core of the model and are also connected to the individuals (rater/manager and ratee/employee) of the PM process. Inputs to the system are everything included in the PM model as well as different contextual factors of relevance. Resources and various organisational strategies and policies can for instance be included as inputs. Outputs are the final outcome of the system and can be exemplified by performance ratings, documentation and feedback, development plans, career planning and/or various recommendations etc. Despite widespread research in the PM field, the evidence-based knowledge of the effects of PM on both organisational and employee level is insufficient [2, 3]. There are also identified knowledge gaps related to all six components of the systems-based taxonomic model [3]. There is for instance an identified need for research focusing on the interaction between employees and managers, but also research focusing on the manager’s interpretation of and role in the PM processes and tasks [3]. Schleicher et al. [3] also state that research focusing on organisational inputs is needed within the field, which corresponds to a described need for research in collaboration with practitioners of specific organisations [2]. Contextual factors are thus considered highly relevant in PM research [2, 3].

For the past few decades an increased focus on performance has also characterised the Scandinavian working life in general [5, 13]. However, in Scandinavia, PM is related to several purposes, such as employee health and well-being, work satisfaction, collegial relations and collaborations, purposes which has been related to working life legislation, strong unions historically, and a general societal norm of equality [13, 14]. In Sweden, it is mandatory for employers to organise yearly manager and employee meetings as part of the Work Environment Authority’s statute collection, to emphasise a good work environment and health [15]. Such dialogues are commonly called “employee reviews”, “job appraisal interviews”, “employee performance appraisals”, “co-worker dialogues”, “staff development talks” or “staff development dialogues” [13]. Due to Sweden’s emphasis on work environment and health, these yearly dialogues have become increasingly focused on how prerequisites for employees’ personal development and well-being can be created in collaboration between employer and employee [13], together with an increased focus on performance [5].

The yearly dialogues can thus facilitate health promotion through participatory and continuous processes focusing on work-related resources and solutions [16]. The workplace has a substantial influence on people’s health and is therefore a prioritised setting for health promotion [17]. A healthy and sustainable working life has become increasingly important, also due to the global challenge of a rapidly ageing population in general [18]. The ageing population is identified as one of the greatest global challenges that will affect society during the next four decades [19, 20]. At the same time another challenge is identified, with many employees leaving working life early due to health problems or disability [20]. It is thus highly important to create opportunities for a healthy and sustainable working life, where employees can contribute and thrive through continuous development of their work-related competence, capacity and performance over the years.

Managers are key actors in workplace health promotion activities/actions, and their approach to such action processes sets the tone and focus [21]. Skilled managers using a health perspective are considered key components of successful workplace health management [22] and managers’ relationships and interactions with employees have a significant impact on employee well-being as well as performance [23, 24]. Managers thus have a key role regarding employees’ prerequisites for workplace-related performance, personal development and well-being through their responsibility for PM, including implementing yearly dialogues with employees. However, dialogues combining employee performance with a focus on employee development and well-being have been shown to be a challenge [13, 23], and research is limited compared to research focusing on traditional PA. More research is thereby needed exploring the complexity of dialogue models combining employee performance with individual development and well-being.

To conclude, working life can benefit from well-functioning PM processes focusing on employees’ development, well-being and health, both from a performance-, a health- and a sustainability perspective. PM is however characterised by complexity in general and especially when combined with focus on employees’ individual development and well-being. A need for research regarding various aspects of PM has been identified, and the importance of knowledge development regarding managers’ interpretation of and role in PM processes and tasks has been emphasised. Another identified knowledge gap relates to contextual factors of relevance for PM, as well as research conducted in collaboration with practitioners. An exploratory study, focusing on managers’ experience of dialogues between managers and employees in practice, was therefore conducted in collaboration with an inter-municipal corporation company in southern Sweden.

## Methods

### Aim

The aim was to explore Swedish managers’ practical experiences of using yearly staff development dialogues (SDDs) to facilitate employee development, performance, and well-being.

### Study design

A qualitative approach with an inductive design [25] was chosen to reflect the aim of exploring Swedish managers’ experiences of using SDDs in practice. Focus group interviews were chosen because they can be used to identify participants’ various experiences and thoughts without the need to reach an agreement on the focused topic [26]. Focus groups also allow participants to develop their original thoughts while communicating with other participants during the interviews, which contributes to a wider understanding of the phenomenon under scrutiny [27]. The interviews were based on a semi-structured interview guide for the gathering of data with a variety of perspectives [28].

### Study context

An inter-municipal corporation company was interested in a research collaboration to explore the use of SDDs in the company. The company was the result of six municipalities merging their previously separate municipal departments dealing with water supply, plumbing and draining. The company had been an inter-municipal corporation company for five years when the study was conducted. The company had a linear organisational structure with a CEO and five departments including both white-collar and blue-collar workers. Each department had its own manager, supported by group managers and supervisors/operating managers. Supportive departments were located at the company headquarters, and included human resources (HR), finance and marketing, among others.

The company´s dialogue model consisted of a yearly cycle that included three separate and formal one-on-one dialogue situations between managers and the employees. This model was based on the company’s values and guidelines, including the three concepts of *work-related joy*, *responsibility* and *competence*. The process started with the SDDs, which were implemented with support from a dialogue guide based on the three concepts listed above. The SDD guide was mainly focused on the employees’ development and performance, although it also included work capacity, wellness, job satisfaction and work-life balance. The SDD prompted employees to share their views on their work situation in general, on the skills of the company management in general, and on the skills of their manager. During the SDDs, individual development goals were set and documented for each employee, based on the development goals of the specific department (for more details see Table 1 below).

**Table 1:** SDD template

1: Theme for the dialogue	Managers preparing comments	Employees preparing comments	Mutual conclusions
Goal achievement/performance and work tasks *The individual goals and tasks, in relation to the goals of the different levels of the organisation* *(7 specific questions)*			
Competence inventory *Activities since previous dialogue* *(single question)*			
Competence and work environment *Opportunities for and initiatives taken to put competence into practice (6 specific questions)*			
Responsibility *The individual in relation to the vision, mission and values of the organisation* *(2 specific questions)*			
Work-related joy *Leadership, co-operation, communication, collegiality, wellbeing and work-life balance* *(7 specific questions)*			
2: Individual development plan			
A: Baseline—main features of the employees work role/tasks	Future challenges	Future demands	
B: Goal setting for individual performance	Activities	Responsible	Timeframe
C: Competence development	Activities	Responsible	Timeframe

The second part of the model was a follow-up dialogue in which the employee’s performance in relation to individual goals was communicated and documented. The third and final part of the model was a salary-focused dialogue related to the performance of the employee. This paper presents the SDD-related findings from the focus group interviews (Additional file 1: Supplementary file 1).

### Participants

All managers (approximately 35) who were in charge of implementing the performance management model at the company were invited to participate in the focus group interviews. Fifteen managers of various ages (12 male and three female) accepted the invitation. These participants represented all five departments of the company and all manager levels. Their professional history and managerial experience varied significantly. Some of the participants had been working as managers for a long time, whether at the previous specific municipal departments or at other companies with different business focuses. Other participants only possessed managerial experience from the study context. Some of the participating managers had previously been employees at the company (Table 2).

**Table 2:** Information about participants

Participant characteristics	(n)
*Sex*	
Male	12
Female	3
*Age*	
25–34	3
35–44	6
45–54	5
55–64	1
*Management level*	
Headquarters	3
Department	5
Group	5
Operational	2
*Years of service in current role*	
< 1	1
1–3	5
4–5	9
*Years of previous managerial experience*	
None	6
< 5	3
5–10	2
10–20	3
> 20	1
*Experience of another role within the company*	
No	10
Yes	5

### Research process

The researcher gave a presentation on the focus group study at a manager meeting, and the managers were provided with written information about the study through the company’s HR department. A total of three focus group interviews were carried out during the winter of 2014–2015. At the start of each interview, the researcher repeated the information about the study orally, and all participants agreed to participate by providing their written informed consent. In order to promote a feeling of openness during the interviews and confidentiality afterwards, participants were reminded of the importance of not sharing each other’s contributions with others afterwards.

The semi-structured interview guide was based on the aim of the study and focused on the participants’ experiences of and thoughts about the company model for PM as a whole. The guide was also inspired by the six components of the systems-based taxonomic PM model: Inputs, Outputs and the four interdependent process factors, namely Tasks, Individuals, Formal and Informal processes [3], which were covered through the recurring support questions: What? How? When? Who? and Why? The interview guide consisted of sets of questions related to the three yearly dialogue situations; the questions asked participants to describe the situations and actions that occurred, with the adherent support questions discuss their own experiences and reflect on contextual aspects, as well as opportunities for improvement (Additional file 1: Supplementary file 1).

Each interview involved four to six participants and one moderator/researcher. The role of the researcher was to initiate the interview and facilitate the conversation by asking open and follow-up questions throughout the rest of the interview. Each interview lasted for 60–75 min and was carried out in a conference room at the company’s headquarters during working hours. The interviews were audio-recorded and transcribed verbatim. A summary of the findings was presented to a small group of participants, who discussed and verified the findings at a meeting in the spring of 2015.

### Analysis

A conventional qualitative content analysis was carried out with the aim of obtaining new insights without using pre-set classifications [29]. The analysis started with the researcher reading the entire body of material a few times, followed by an initial coding. A condensation was then made, concentrating on the SDDs under scrutiny in this paper. The second reading phase focused on the identification and coding of meaning units; next, the researcher read through the entire body of material again. Codes representing similar phenomena were then combined into subcategories, and the subcategories were compiled into three categories based on their related content. Examples from the step-by-step process of analysis is presented in Table 3, also showing the three categories emanating from the analysis.

**Table 3:** Examples from the step-by-step process of qualitative content analysis

Meaning unit	Code	Subcategory	Category
If you know that there will not be any changes, this position will not develop. Because that’s the way it is sometimes	Opportunities for development in the company	A natural part of the company	SDD in a business context
The SDD is a concrete tool for pushing things forward. The purpose is development of the individual as well as the department. So for me, concretisation and clarity are important	Instrumental style	Manager approach	Managers in relation to SDD
I notice a big difference. When I was a new manager, the SDDs were much shorter. The co-workers didn’t know me then. Now they have begun to open up and tell me more about their situation at home, and so on. It’s a confidence that I’m trusted with, and hopefully it will increase	Relation between employees and manager	Confidence and trust	Employees in relation to SDD

The author (ÅB) performed the step-by-step analysis process, with the co-author (PNL) functioning as a critical dialogue partner with access to the data throughout the process. The analysis was thus conducted in collaboration between the authors.

## Results

The findings include a range of managers’ experiences with and thoughts on the company’s SDDs, which were sorted in three categories with adherent subcategories (see Table 4 below).

**Table 4:** Subcategories and categories that emanated from the analysis

Subcategories	Categories
The content and the process	SDD in a business context
Goal setting	
A natural part of the company	
Usefulness	Managers in relation to SDD
Resources	
Manager approach	
Approach and motivation	Employees in relation to SDD
Confidence and trust	

The categories and subcategories that were identified during the analysis are used as subheadings below and the findings are complemented with quotes from the focus group interviews throughout, for example F2 in brackets means focus group number two.

### SDDs in a business context

The participating managers related their experience of SDD to the business context with a specific SDD model. These findings were organised with the subcategories: *The content and the process; Goal-setting;* and *A natural part of the company*.

#### The content and the process

The content of the SDDs was established on company level when the company was formed and was accessible for employees through the company intranet. As a result, the content was well known among the employees, resulting in good opportunities for both the managers and the employees to prepare themselves for and conduct the SDD.

The participating managers discussed the suitability of the SDD guide they were given, in relation to various parts of the company, and often described it as being more suitable for SDDs between managers and with white-collar workers. Some of them therefore expressed a wish for an SDD template with a stronger connection to the specific work situations of employees in different parts of the company.

They [the employees] feel that the questions aren’t directed towards them. Maybe that could be changed in order to get their attention. I think that they want the instrument to be more directed towards the technical aspects of what they are doing.

You are right, our units are very different. My workers never talk about the technical aspects of their work. (F2)

Other participants thought that the SDD guide made it possible for them to adapt the dialogue to a specific situation and employee.

I think it’s a good template. Then it’s up to you to build the dialogue from it. (F2)

However, the managers commented that the employees often expected the managers to follow the SDD guide in detail, which limited the possibility of adapting it for the employee and specific SDD situation at hand.

It’s like, if I ask a question that is not the exact same wording as in the template, they say “where is that question, which page are you on?” (F3)

The findings also showed the importance of a logic structure for the SDD process at company level. The managers supported the existing top-down process, in which the company business strategy for the following year was the first step of a goal-setting procedure including all the levels of the company.

#### Goal-setting

The next step of the goal-setting process was department-based goal-setting during the departmental managers’ SDDs with the CEO. The third step involved departmental managers having SDDs with their group managers, during which group-based goal-setting was carried out, and so forth. In the last step of the process, individual goals were set for each employee during an SDD with their manager. This process was characterised by the decomposition of the business strategy into specific goals for each part of the company and each manager, and into individual goals for each employee. The managers participating in the focus group interviews continuously came back to the concept of goal-setting. They described experiencing difficulties in relation to the decomposition of goals from one level to another, especially in relation to the concretisation and measurability of individual goals for employees in different parts of the company.

We have an SDD and try to relate it to the business plan of the company. Breaking it down to each unit is difficult, so individual goal-setting must be even harder. (F1)

No matter how specific and measurable you want it to be, it is very hard to be really specific and measurable. (F3)

Most of the participants considered that goal-setting needed to result in concrete and measurable goals in order for the SDD to function as a good basis for following up on the performance and development of employees, groups and departments.

If you don’t have these measurable goals, there is just a lot of guessing and thinking at follow-up. (F3)

#### A natural part of the company

The findings showed that the overall aim of the company’s performance management model was continuous development of the staff and thereby also development of the work groups, the departments, and the company. According to the participants, this focus on development was a conscious strategy that was established after the merger of the inter-municipal company and the reason for using the concept SDD for the model.

The participants described a complex company with a variety of operational activities along with associated work situations and tasks for employees. This variation was related to an experienced challenge with implementing SDDs with the same development aim throughout the company. The participants considered that there were numerous work situations in which the possibilities for development were very limited, particularly for blue-collar workers.

If you know that there will not be any changes, this position will not develop. Because that’s the way it is sometimes. (F1)

Therefore, the strategy to focus on development throughout the company was sometimes questioned.

The managers stressed the need for a company culture of feedback in order for the SDD model to function well. An everyday practice in which the interactions of employees and/or managers were characterised by an open climate with both positive and negative performance-related feedback was related to the SDD becoming widely accepted and a natural part of manager/employee interaction.

The SDD needs to be widely accepted , you know. It needs to be built in, like a natural part to think about throughout the year. We need to be used to and have engaged and open discussions with our managers at work, where we can say what we think. (F2)

The participating managers considered that the company culture needed to be based on a common view of the SDDs’ usefulness and importance, with the accompanying attitudes and behaviours of taking it seriously.

### Managers in relation to SDDs

As mentioned earlier, the managers were responsible for the SDDs and were part of the systematic implementation of the SDD process at the company. From the managers’ perspective, the SDDs were related to the subcategories: *Usefulness; Resources;* and *Manager approach.*

#### Usefulness

The SDDs were generally considered useful to the managers in their work. However, various managers described this usefulness rather differently. Some referred to the SDDs as an important development-focused complement to their more practice-based everyday interactions with employees. Others described SDDs as a useful practice-focused complement to their work, as they did not have enough time to interact with their employees on an everyday basis. The managers thus positively described the secluded sit-down dialogue with their employees during the allocated time.

You don’t have time otherwise, so, at least once a year, you can sit down during allocated time for dialogue in privacy. (F1)

Employee feedback during SDDs was considered to help the managers to improve their leadership skills. The participants appreciated the feedback they received during SDDs but remarked that the employees had difficulty giving negative feedback on their manager’s leadership skills.

There must be many situations throughout a year where I have done something wrong, things that can be improved. It’s just so difficult; they all say everything’s just fine, which is good feedback, but it’s very difficult to bring out the negative. (F2)

#### Resources

The findings showed that well-functioning and useful SDDs needed resources from both a structural and a personal managerial perspective. The managers emphasised the importance of allocating enough time for SDD preparation and execution. However, finding that time could be challenging in a stressful work situation. The findings also highlighted a need for managers to have the authority and power to act in accordance with the result of the SDD.

I sometimes wish that I had more authority, when I’m talking to employees with ambitions, you know. (F2)

Knowledge of the employees, their work and the work situation was also considered to be a resource for managers in carrying out SDDs. Knowing the employees was strongly related to the need for the managers to adapt the SDD to the employee’s situation – a key aspect of successfully carrying out SDDs throughout the whole company.

You have to adapt according to who you are talking to and the situation they are in. (F1)

#### Manager approach

The participating managers acknowledged their own role in SDDs and commented that their experience, personality and so forth affected their execution of SDDs in different ways. The managers’ interaction in the focus groups revealed two managerial approaches towards SDDs: *instrumental* and *relational* approaches. These approaches showed no direct link to sex, age or manager experience of the participants. Participants who gave a rather technical and delimited description of SDDs in general were categorised as using the instrumental approach.

The SDD is a concrete tool for pushing things forward. The purpose is development of the individual as well as the department. So for me, concretisation and clarity are important. (F3)

Other participants described SDDs as being more interpersonal and holistic, and they were categorised as using the relational approach. This approach included getting to know the employee and talking about aspects of well-being.

I find it really nice to sit down once a year – sometimes I wish it was more often – and really talk about how they [co-workers] are doing, what they are doing, how things are working and so on. (F3)

### Employees in relation to SDD

The SDDs were strongly focused on the employees since, in practice, an SDD was a dialogue with employees about them and their work situation. The findings included in the category were structured under the two subcategories: *Approach and motivation;* and *Confidence and trust.*

#### Approach and motivation

The participating managers described how employees showed different attitudes and behaviour towards the SDDs. For example, some employees were hardly prepared for the SDD at all, while others took notes throughout the year and were very well equipped for the SDD. Besides variety in preparation, the findings exposed a variety of employee attitudes to SDDs in particular, and to everyday practice and development in general. The participants commented that some employees were more focused on organisational aspects related to their everyday practice than they were on their own role, performance and development. The findings indicated that it was more common for blue-collar workers, in comparison with other employees at the company, to focus on aspects of their everyday practice than on development.

The attitude among [blue-collar] co-workers, I hear…they say, “I just want to do my job, I don’t want to make plans on what to develop or how to relate to other parts of the company. I just want to go out and do the job, you know”. (F1)

Some of the managers also mentioned that a focus on development decreased with age among the employees. Thus, it was difficult for the managers to achieve a focus on development throughout the company, in relation to the execution of SDDs.

The participants emphasised the importance of the employees being aware that they were part of a larger whole and that their development and performance thus contributed to performance on group, department, and company levels. The managers considered that this perspective ought to act as a motivation for employees to develop their performance. They also stressed, however, that this motivation could not be taken for granted, since the employees cared to greater or lesser degrees – or sometimes not at all – about the company as a whole.

To see the goal in 2035 for the company, for instance, doesn’t motivate everyone. Maybe I’m [a co-worker] more focused on doing a really good job here and now. Then I go home, and that’s it.

Yeah, but I really hope that the majority of the co-workers want to know about why we are doing this or that. That it motivates them and they perform better at work. (F1)

The findings revealed that the managers preferred an employee commitment towards development and discussion during the SDDs. They also stressed that when the employees took the SDDs seriously and were committed to development, the employees then had higher expectations and the managers were required to exert more effort and prepare themselves more for the SDDs.

The participants experienced some employees to be more willing to communicate on a personal level, while others were considered more task-oriented. Some of the managers highlighted an advantage of SDDs that were more personal, since they provided a better opportunity to get to know and discuss the well-being of the employee.

You have time to find out how they [the co-worker] are doing and feeling, how it’s been during the year. I have learned a lot from talking about how they feel in general during SDDs, even though I talk a lot with them on an everyday basis. Things you don't see otherwise in the business. (F3)

#### Confidence and trust

The participants continuously returned to the aspects of confidence and trust in their employee relations, which they described as crucial for a well-functioning SDD.

I notice a big difference. When I was a new manager, the SDDs were much shorter. The co-workers didn’t know me then. Now they have begun to open up and tell me more about their situation at home, and so on. It’s a confidence that I’m trusted with, and hopefully it will increase. (F2)

Confidence and trust were thus considered to take time to build, and to vary depending on the relational character of the specific employee-manager interaction.

The specific employee-manager interaction, including the employees and managers relation and approaches to SDDs, was considered to be the core of the complexity surrounding SDDs in practice. The character of SDDs could thus vary, even though each was conducted with the same template, within the same process, with the same formal aim and in the same company.

## Discussion

This exploratory study revealed a variation in Swedish managers’ experiences of using SDDs, in practice, at an inter-municipal corporation company. The participating managers’ practical experiences, analysed with the three categories: *SDD in a business context; Managers in relation to SDD;* and *Employees in relation to SDD*, can to a large degree be understood as different types of determinants of SDDs in practice. For example, the categories of *Employees* and *Managers in relation to SDD* can be linked to [3] an interpersonal perspective, in which interaction and communication act as the core of the SDD model at the company. Placing human interaction at the core of a process aiming for human development, performance and well-being at work can thus be recognised from the systems-based taxonomic model of PM [3], but also from the systems-based Organisational Health Development (OHD) model, which shows the complex interaction between personal and organisational health determinants and processes [30]. The OHD model shows the mutual relationship between individual capabilities, such as competence, motivation as well as identity, and organisational factors represented by structure, strategy and culture. The model also includes task-related processes, including business and management as well as relational aspects and processes, such as leadership, relations and discourse between managers, employees and eventually third parties, such as customers. The findings also show that business-related factors, such as the company-specific context and SDD model, can be related to the managers’ experience of SDD in practice. Contextual factors are also included in the OHD model [30] and are considered inputs in the taxonomic model for PM by Schleicher et al. [3]. Contextual influence on the process and outcome of employee performance has been identified as one of the most important research contributions within the field [2].

Prerequisites for individual performance include a general knowledge of the organisation’s strategic goals, sound human resource procedures, and efficient PM [2, 3]. The participating managers communicated a taken-for-granted viewpoint—that development of performance on the individual level leads to improved performance on the organisational level—and therefore expressed a preference for employees who were motivated towards development. However, research has shown challenges in confirming meaningful links between the individual and organisational levels, and there is a need for research focusing on how improving performance at the individual level can enable improvement in organisational performance [2]. Efficient performance systems have previously been related to effective system designs, effective managerial system practices and effective appraisal support [10]. The findings indicate that the SDD model of the company can be considered a model for PM since the model aims for organisational development/improvements and is characterised by a cyclic and action-oriented organisational process [2, 3]. The model also includes employee performance assessment and improvement through goal-setting, appraisal and feedback [2, 3].

The findings of the present study highlight the importance of goal-setting when aiming for employee development and performance through SDD, which aligns with setting performance expectations in PM [3] and PA [2, 10]. The participating managers supported the existing top-down goal-setting procedure in the SDD process at the company level, with a step-by-step breakdown of the company business strategy into measurable goals for each department and further down into measurable individual development goals for each employee. Previous research has shown that performance rating and goal setting facilitates dialogue concerning performance improvement [4, 10] and can be powerful management tools, which can improve employee reactions and performance [4]. However, the participating managers experienced difficulties in relation to goal-setting as part of the SDD model, indicating a need for support and competence development in goal-setting. The need for communication and management support has previously been identified in relation to co-worker dialogue and to PA [10, 23]. Education and training in goal-setting and performance rating have, thus, been found to be crucial for an efficient PA process [10, 23] and experience of well-functioning PA facilitates a positive relation between assessment, job satisfaction and motivation [4, 12].

The experience of the participating managers showed that employee motivation and engagement in development and SDD varied, and that the development focus of the SDD used at the company was more suitable for white-collar workers. Prior research has also shown that employees with higher education are more likely to experience increased job motivation due to PA [31], but also that PA without sufficient employee benefits can have a negative effect on purposeful and dedicated employees [4]. Previous research thus supports the participating managers’ questioning of the general focus on development through assessment for all employees at the company. The experience of the managers revealed a business complexity, with variation between different parts of the company, and adherent importance of constantly adapting the SDD to the employees for promotion of their motivation and engagement in the SDD process. Research have shown that leadership adaptation to the specific needs of employees helps employees thrive [22] and strategies that facilitate employees’ engagement and participation at work provide an opportunity to increase the employees’ understanding of their own role in relation to the entirety of an organisation, leading to useful development of the organisation [32].

Strengthening the participatory parts of the SDD model can thus have a positive effect on employees’ motivation for development in relation to the company, which is in line with the managers’ preference for development-motivated employees. Strengthening the participatory parts of the model, however, also makes it possible to reinforce a health perspective of the SDD, and provides an opportunity for more efficient health management on company level. Participation is a key component within the field of workplace health promotion (WHP) [33] and the yearly dialogues can promote health through a participatory and systematic process concentrating on work-related resources and solutions [16, 22].

The collaborative involvement of various stakeholders for health is a prerequisite for WHP, as well as sustainable workplaces [18, 34]. Sustainable workplaces can lead to better fulfilment of the employer’s health-related obligations according to Swedish legislation on systematic work environment management [15, 35]. A lack of compliance with employers’ obligations has previously been identified among Swedish employers [36], and managers’ role and approach have been acknowledged in relation to well-functioning workplace health initiatives [21, 22, 24].

The findings indicate that when managers use an instrumental approach there is a strong focus on development at the organisational level and the SDD is less likely to have a health or well-being perspective, which can be recognised from the criticism of dialogue models like PA [7, 9]. However, in Scandinavia PM is often combined with a focus on the well-being of the employees, due to the working life legislation [13, 14], and has previously been identified as a challenge but nevertheless a tool for improving employee health and for fostering a development culture in the workplace [23]. According to the findings, a well-being perspective in SDDs is facilitated by managers using a relational approach, employees being open to discussing personal matters and an employee-manager relationship that is characterised by confidence and trust.

Given that the findings of the present research identified managers who used an instrumental rather than a relational approach, and some employees being more task-oriented than personal, the well-being perspective within the SDD process is probably more of a coincidence than a conscious and systematic part of the process. Thus, from a well-being perspective, the SDD process seems to be a missed opportunity for WHP, a whole-system approach that combines the employees’ individual development with their experience of health [34]. WHP has, however, also been criticised for violating employee boundaries in terms of culture, privacy, confidentiality and ethics, based on WHP resulting in a medicalisation of working life [37]. Traditionally WHP has also been characterised by a criticised behaviour change approach [38], which can be related to difficulties in distancing health promotion from the traditional medical health model [39]. From an ethical point of view the empowerment model is considered better [38], which corresponds well with WHP as a settings approach [39, 40], and might be a way to reduce a negative effect of the identified problematic power imbalance between managers and employees in WHP [37].

The participating managers’ experiences highlight the need for resources, enabling managers really knowing their employees and having the time but also the power to act in accordance with the outcome of the SDD for a motivational and useful SDD. The findings showed that the managers experienced the SDD as useful regarding promoting employee development, performance and well-being, but also for the opportunity to receive feedback on their leadership from the employees. The SDD can thus have a positive impact on the manager-employee relation and offers an opportunity for increased loyalty and work engagement [4, 5, 14]. Along with motivation, health plays a significant role in the complex interactions between individuals and organisations [30]. Managers thus have a key role in their responsibility for PM, including implementing yearly dialogues with employees, and employees’ prerequisites for workplace-related performance, personal development and well-being.

### Methodological considerations

The methodological considerations are presented with support from the framework of trustworthiness in qualitative inquiry [41]. In order to strengthen the study trustworthiness with focus on conformability, a summary of the findings was presented to and verified by some of the participants. The researchers also continuously discussed the findings throughout the process of analysis. The trustworthiness of this work was also strengthened by presenting the description of the analysis process, together with examples from the step-by-step process of analysis (Table 3), and the presented findings in combination with quotes from the interviews.

Focusing on managers exclusively can be considered a weakness since SDDs are conducted for and with employees, which makes the employees’ experiences important to explore. However, the focus on managers exclusively made it possible to explore SDDs in practice, in terms of their specific role and responsibility, which was in line with an identified research priority within the PM field [3] and thereby also a delimitation in the aim of the study. An in-depth study, focusing on the employees’ experiences exclusively, can instead be conducted with support from the findings of this study. The use of participants from only one company, in which everyone used the same SDD model, process and template for performance management, can be considered as both a weakness and a strength. It is a weakness in that it made this study context-specific and thus set limitations on transferability, but it is a strength in that it permitted greater exploratory depth and authenticity. Individual interviews would probably have generated even more depth regarding, for instance, the doubts and challenges faced by the participants. However, using focus groups provided useful insights related to the general social/cultural practices. The smallness of the study can also be considered a weakness, and the trustworthiness of the findings would have been strengthened if more managers from the company had participated in the study.

A thorough description of context has been included in order to facilitate contextual understanding for readers. The contextual variation within the company and the participants’ differences in age, sex, managerial experience, departments and management levels within the company strengthened the heterogeneity and transferability of this work, even though this study was context specific. The variety of the findings can be considered a verification of the heterogeneity of the participants; it also indicates that the focus group interviews supported the participants in sharing their differing thoughts about SDD performance.

## Conclusions

The findings of the study add to the literature on the complexity of performance and health management and can be used as a framework for understanding and improving performance and health management through yearly dialogue processes in working life. The findings revealed opportunities as well as challenges involved in PM in practice. Education and training in dialogue method were shown to be needed, as well as communication and reflection among manager colleagues, to support managers in their responsibility for conducting SDDs. The study also shows how a well-being perspective seems to be more of a coincidence than a systematic and natural part of the SDDs, due to the link between well-being and the manager-employee relationship as well as approaches to SDDs. Strengthening the well-being perspective through systematisation and participation will thus improve the quality of the SDD model, which in turn can facilitate sustainable workplaces and better fulfilment of employers’ health-related obligations for systematic work environment management. More research is needed regarding the complex phenomenon of PM in practice and especially concerning the challenges in aiming for employee development, performance as well as well-being through yearly dialogue processes between managers and employees.

## Supplementary Information

**Additional file 1:** Semi structured interview guide used in the focus groups.

## Data Availability

The datasets generated and analysed during the current study are not publicly available as a whole, due to the confidentiality agreement with the participants. Parts of the data can, however, be available from the corresponding author on reasonable request.

## Abbreviations

OHD: Occupational health development model
PA: Performance appraisal
PM: Performance management
SDD: Staff development dialogue
WHP: Workplace health promotion
