# Supplementary material for: Swedish managers’ experience of yearly staff development dialogues, aiming for employee development, performance, and well-being
Source: BMC Psychol. 2022 Jul 27;10:184. doi: 10.1186/s40359-022-00890-w (PMC9327207; doi:10.1186/s40359-022-00890-w)
Supplement: Supplementary file 1 — Additional file 1: Semi structured interview guide used in the focus groups. [file 40359_2022_890_MOESM1_ESM.docx]

Supplementary file 1: Semi structured interview guide used in the focus groups.

| **Part of the Performance Management (PM) model** | **Question areas** |
| --- | --- |
| *****The PM model as a whole | - Description of the model - Experiences in general - Functionality and opportunities for improvement |
| *****Staff Development Dialogue (SDD) | - Description of the dialogue (what, how, when, who, why) - Experiences in general and contextual aspects in particular - Functionality and opportunities for improvement |
| Follow-up dialogue | - Description of the dialogue (what, how, when, who, why) - Experiences in general and contextual aspects in particular - Functionality and opportunities for improvement |
| Salary-focused dialogue | - Description of the dialogue (what, how, when, who, why) - Experiences in general and contextual aspects in particular - Functionality and opportunities for improvement |

***** Data emanating from these parts of the focus group interviews represents the unit of analysis in this article.
